# Exosome-Encapsulated miR-31, miR-192, and miR-375 Serve as Clinical Biomarkers of Gastric Cancer

**DOI:** 10.1155/2023/7335456

**Published:** 2023-02-16

**Authors:** Jing He, Jing Wu, Shiyang Dong, Jing Xu, Jian Wang, Xin Zhou, Zhuqing Rao, Wen Gao

**Affiliations:** ^1^Department of Oncology, The First Affiliated Hospital of Nanjing Medical University/Jiangsu Province Hospital, Nanjing, China; ^2^Department of Anesthesiology, The First Affiliated Hospital of Nanjing Medical University/Jiangsu Province Hospital, Nanjing, China

## Abstract

In recent years, microRNAs (miRNAs) derived from exosomes have been attracting attention as novel clinical biomarkers in a variety of cancers. In this study, plasma samples from 60 gastric cancer (GC) patients and 63 healthy individuals were collected, and the exosomal microRNAs (ex-miRNAs) were isolated. We determined the specific ex-miRNAs through miRNA microarray and a database of differentially expressed miRNAs called dbDEMC. Then, the expression levels of exosomal miR-31, miR-192, and miR-375 were analyzed by quantitative polymerase chain reaction (qRT-PCR). Compared to the matched controls, exosomal miR-31, miR-375, and miR-192 were significantly upregulated in GC patients. Also, they were found to be associated with gender, with miR-192 being significantly upregulated in male GC patients. Kaplan–Meier analysis indicated that high expressions of exosomal miR-31, miR-375, and miR-192 were positively correlated with poor clinical outcomes of GC patients. Cox univariate and multivariate analysis found that ex-miR-375 expression and TNM stage were independent prognostic factors of overall survival (OS). Our findings revealed that exosomal miR-31, miR-192, and miR-375 might serve as noninvasive, sensitive, and specific biomarkers for the diagnosis and prognosis of GC patients.

## 1. Introduction

Gastric cancer (GC) has become the third cause of cancer-related death and one of the most commonly diagnosed malignant cancers worldwide [1]. The five-year survival of advanced GC patients is less than 7%, while the median overall survival (mOS) is less than 1 year [2]. The development of GC treatment such as endoscopic resection and surgery has markedly improved the prognosis of early-stage GC patients. However, symptoms of GC often appear in an advanced or metastatic stage, resulting in poor clinical outcomes and high mortality rate. Therefore, diagnostic and predictive biomarkers are urgently needed for GC patients. Noninvasive and accurate detection markers are necessary to improve the long-term survival of GC patients through timely therapeutic interventions and disease management [1].

MicroRNAs (miRNAs) are one kind of small noncoding RNAs capable of modulating gene expression via RNA interference at the posttranscriptional level [2]. Specific miRNAs play crucial roles in multiple processes of carcinogenesis, such as cell proliferation, autophagy, migration, and invasion [3]. These miRNAs have been found to be extremely stable in the plasma exosomes and protected from endogenous RNase activity [4]. Thus, exosomal miRNA (ex-miRNA) could serve as a highly sensitive and specific tool suitable for cancer screening. Exosomes are microvesicles secreted by various living cells, including mastocytes [5], platelets [6], cancer cells, and other cells which contain multivesicular endosomes [7]. After multivesicular compartments fusion with the plasma membrane, exosomes can be secreted to the extracellular environment [8] and function as carriers of miRNAs, DNAs, and proteins to mediate intercellular communication [9, 10]. Exosomes are also reported to participate in many vital bioactivities, such as cell differentiation, angiogenesis, antigen presentation, and immune response [3].

Increasing evidence showed that cancer cells might express higher levels of specific ex-miRNAs compared to normal controls in a large amount of human cancers such as esophageal squamous cell cancer (ESCC) [4], prostate cancer [11], and lung cancer [12]. Therefore, we hypothesized that some ex-miRNAs would be increased in the plasma of GC patients. We obtained the miRNA profiling from a gastric adenocarcinoma dataset from the Database of Differentially Expressed MiRNAs in Human Cancers (dbDEMC). In combination with our previous screening study using miRNA expression microarray data (data not shown), three miRNAs including miR-31, miR-192, and miR-375 were selected. Then, these miRNAs encapsulated in exosomes were extracted from patient plasma and further examined with quantitative real-time polymerase chain reaction (qRT-PCR) analysis to measure their expression levels. Finally, we found the expression levels of exosomal miR-31, miR-192, and miR-375 were significantly increased in GC patients compared to healthy donors.

Previously, miR-31, miR-192, and miR-375 have been reported to participate in various processes of tumorigenesis and progression in several cancers, such as colorectal cancer [13], breast cancer [14], lung cancer [15], and others. However, there is limited literature demonstrating the potential relationship between these three miRNAs encapsulated in exosomes and gastric cancer progression. In the present study, we explored the biological role and clinical significance of exosomal miR-31, miR-192, and miR-375, in order to provide a new insight into promising indicators for early diagnosis and prognosis prediction for GC patients.

## 2. Materials and Methods

### 2.1. Patients and Plasma Samples

Sixty GC patients and 63 healthy donors (as normal controls) were enrolled at the First Affiliated Hospital of Nanjing Medical University from 2014 to 2016. The survival time of all patients was defined as the duration from the diagnosis date to the follow-up deadline, December 31, 2020. We obtained the blood samples under the approval of Institutional Ethical Committee. Patients did not receive any treatment intervention such as surgery, chemotherapy, or radiotherapy. Tubes with ethylenediaminetetraacetic acid (EDTA) (BD, New Jersey, USA) were used to collect blood samples from patients and healthy donors. Plasma samples were isolated from blood within 6 hours. Samples were clarified by spinning at 350 relative centrifugal force (RCF) at 4°C for 10 min and 20,000 RCF for 10 min.

### 2.2. Isolation and Identification of Exosomes

According to the instructions of the manufacturer (System Biosciences, Mountain View, CA, USA), exosomes were extracted carefully from plasma samples. The 200 *μ*L plasma was mixed with 100 *μ*L ExoQuick exosome precipitation solutions and centrifuged continuously. To identify the existence of exosomes, the sediment was diluted in phosphate buffer saline (PBS) at room temperature. Subsequently, exosomes were stained with a drop of 2% phosphotungstic acid for 2 min and observed under a transmission electron microscope (TEM, JEM-1010 microscope, Japan).

### 2.3. Western Blot Analysis

To evaluate the efficacy of exosome separation, we examine the characteristics of exosomes with Western blotting. Briefly, pellets of exosomes were collected, and the RIPA lysis buffer was utilized to isolate exosomal proteins. The bicinchoninic acid assay (BCA) method was performed to evaluate the quantification of proteins. Proteins were loaded onto sodium dodecyl sulfate-polyacrylamide gel (SDS-PAGE) for electrophoresis and transferred to a polyvinylidene fluoride (PVDF) membrane. After blocking with bovine serum albumin (BSA) for 2 h, primary antibodies CD63, CD9, and TGS101 (BOSTER, 1 : 1000) were prepared to incubate the membrane for 24 h. Subsequently, the HRP-conjugated secondary antibody was incubated with the membrane for 2 h. Finally, transferred proteins were detected by chemiluminescence with a ChemiDoc XRS + Imaging system (Bio-Rad Laboratories, Inc., Hercules, CA, USA).

### 2.4. RNA Extraction and qRT-PCR

Total RNAs (including miRNAs) derived from exosomes were extracted by a MirVana Paris Kit (Ambion, Austin, TX, USA) according to the manual. Denaturing solution (Ambion, Austin, TX, USA) was added to each sample. Total RNAs were dissolved in 100 *μ*L RNase-free water. For further measurement, samples were stored in a −80°C refrigerator. The concentration of RNAs was assessed by a Nanodrop 2000 spectrophotometer (NanoDrop Technologies, Wilmington, DE, USA). After that, reverse transcription was applied to synthesize cDNA using the TaqMan Micro-RNA Reverse Transcription Kit (Thermo Fisher Scientific). The expression of ex-miRNAs was quantified by qRT-qPCR analysis according to the instructions of the manufacturer. We examined the obtained cDNA by qRT-PCR with TaqMan microRNA primers specific for miR-31, miR-192, and miR-375 (Thermo Fisher Scientific Inc., Waltham, MA, USA). A 7900HT real-time PCR system (Applied Biosystems, Foster City, CA, USA) was employed for amplification and evaluation for miRNAs extracted from the exosomes. The miRNA expressions were calculated by 2^−ΔΔCt^ relative to RNU6B (U6): ΔC_t_ = C_tmiRNA_ − C_tU6_.

### 2.5. Statistical Analysis

The Mann–Whitney test was used to assess the statistical significance of differentially expressed miRNAs in GC. *χ*^2^ test or one-way ANOVA was utilized to explore the relationship between ex-miRNAs and clinical characteristics. Furthermore, the evaluation of sensitivity and specificity of each index was relied on receiver-operating characteristic (ROC) curves and the area under the ROC curve (AUC) for GC detection. Finally, overall survival (OS) was examined with the Kaplan–Meier survival curve method, and the resulting data were examined using logrank and Wilcoxon tests. A Cox proportional hazard regression analysis was applied to estimate the univariate and multivariate hazard ratios of survival. We used SPSS 20.0 software (SPSS Inc., Chicago, IL, USA) and GraphPad Prism 7.0 (GraphPad Software, USA) to perform statistical analyses and graph plotting. If a *P* value <0.05, it is considered significantly different.

## 3. Results

### 3.1. The Characteristics of Patients

The present study enrolled 123 volunteers, including 60 GC patients and 63 healthy controls.  Table 1 shows the detailed clinical characteristics of these recruited participants. It is worth noting that the gender and age distribution between GC patients and healthy controls had no statistical difference.

### 3.2. Identification and Characterization of Exosomes

**Figure 1:**
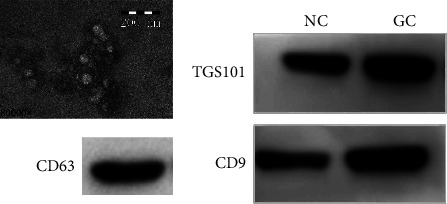
Confirmation of exosomes extracted from plasma samples with TEM to ensure exosomal morphology and size distribution (upper; original magnification 80,000x), and exosomal markers CD63, CD9, and TGS101 by Western blot analysis (below; the blot image was cropped from the full gel detecting CD63 from different samples). TEM: transmission electron microscopy.

In order to identify exosomes derived from plasma samples, TEM was utilized, and indicated exosomes presented a circular vesicle with a diameter of about 100 nm (Figure 1(a)). We applied Western blotting to identify the isolation of exosomes using exosomal protein markers CD63, CD9, and TGS101 (Figure 1(b)). The results indicated the successful separation of exosomes from blood samples.

### 3.3. Prediction and Identification of the Targeted miRNA Profile in GC Patients

In order to determine the targeted miRNAs, we collected abundant data on the potential miRNAs expressed in GC patients taken from a public miRNA-target database called dbDEMC (https://www.biosino.org/dbDEMC/index). After that, we combined the collected information with our miRNA expression microarray data (data not shown) to identify the differentially expressed miRNAs in gastric cancers compared to healthy controls. Finally, we regarded miR-31, miR-192, and miR-375 as potential biomarkers for the diagnosis and prognosis of patients with GC (see Supplementary 1 and 2 for details).

### 3.4. Expressions of Ex-miRNAs Were Significantly Higher in GC Patients

**Figure 2:**
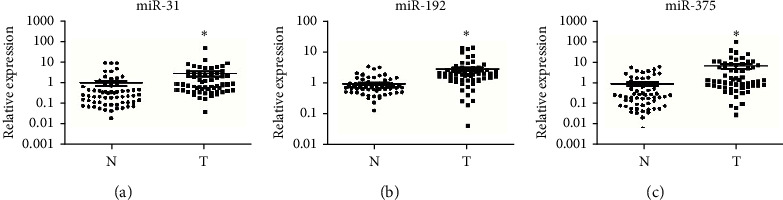
Expression levels of plasma exosomal miR-31, miR-192, and miR-375 in 60GC patients and 63NCs. (N) normal control; (T) tumor. Horizontal line: mean with 95% CI. ^*∗*^*P* value <0.05.

The levels of exosomal miR-31, miR-192, and miR-375 collected from plasma samples were compared between 60  GC patients and 63 matched controls. By normalization to U6, the results revealed that all of these three ex-miRNAs were significantly higher in GC patients than in healthy controls (with a fold change of miR-31 for 2.85, miR-192 for 2.29, and miR-375 for 4.85; Figure 2).

### 3.5. Sensitivity and Specificity of Ex-miRNAs as Diagnostic Biomarkers

**Figure 3:**
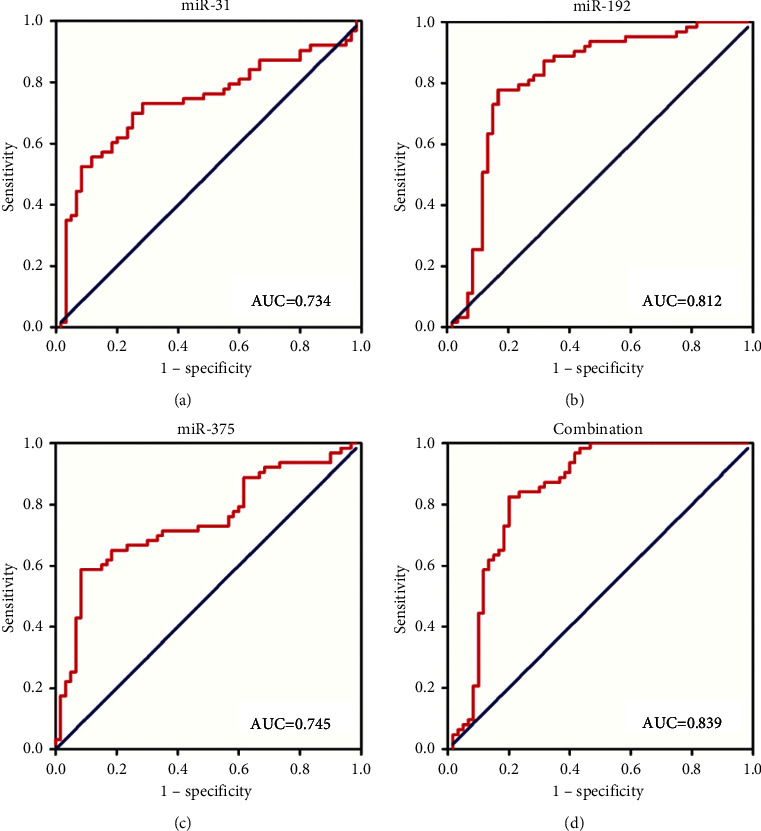
Receiver-operating characteristic (ROC) curve analyses of exosomal miR-31 (a), miR-192 (b), miR-375 (c), and the combination of the three miRNAs (d) to differentiate GC patients from normal controls. AUC: areas under the curve.

Qualified biomarkers possess adequate sensitivity and specificity. We generated ROC curves based on the expression of ex-miRNAs and calculated AUCs to estimate the sensitivity and specificity of exosomal miR-31, miR-192, and miR-375 for GC detection. As presented in Figure 3, the AUCs for miR-31, miR-192, and miR-375 were 0.734 (95% confidence interval (CI) = 0.643–0.826), 0.812 (95% CI = 0.730–0.895), and 0.745 (95% CI = 0.656–0.833), respectively. Furthermore, we also identified the AUC of the miRNA panel (combining exosomal miR-31, miR-192, and miR-375 together) was 0.839. The equation for the panel is as follows: Logit (P) = 0.376 + 0.079 *∗* miR-31 + 0.791 *∗* miR-192 + 0.19 *∗* miR-375. According to our results, expressions of these three plasma ex-miRNAs, including the ex-miRNA combining panel, could distinguish GC patients from the healthy population and act as promising diagnostic biomarkers.

### 3.6. Relationship between Ex-miRNAs and Clinicopathological Factors

The 60 GC patients were separated into two groups based on the intermediate values of three ex-miRNAs as tangent points. All patients were split into high miR-31 expression group and low miR-31 expression group, and the same as other two ex-miRNAs groups. The relationship between clinicopathological characteristics and expressions of exosomal miR-31, miR-192, and miR-375 is presented in Table 2 (presented as *P* value). A statistically significant association was observed between the expression values of these three ex-miRNAs and age. Interestingly, ex-miRNA was highly expressed in younger individuals (age <65) than elder individuals (age ≥65) (with a fold change of 1.95 for miR-31, *P*=0.008, fold change of 2.10 for miR-192, *P*=0.005, and fold change of 1.96 for miR-375, *P*=0.05, respectively). Moreover, only miR-192 was significantly upregulated in male GC patients than in females (with a fold change of 2.72, *P*=0.036). There was no significant difference in other clinicopathological factors, including tumor location, neural invasion, vascular invasion, Lauren classification, and TNM stage (all *P* > 0.05).

### 3.7. Expressions of Ex-miRNAs Were Associated with the Survival Rate of GC Patients

**Figure 4:**
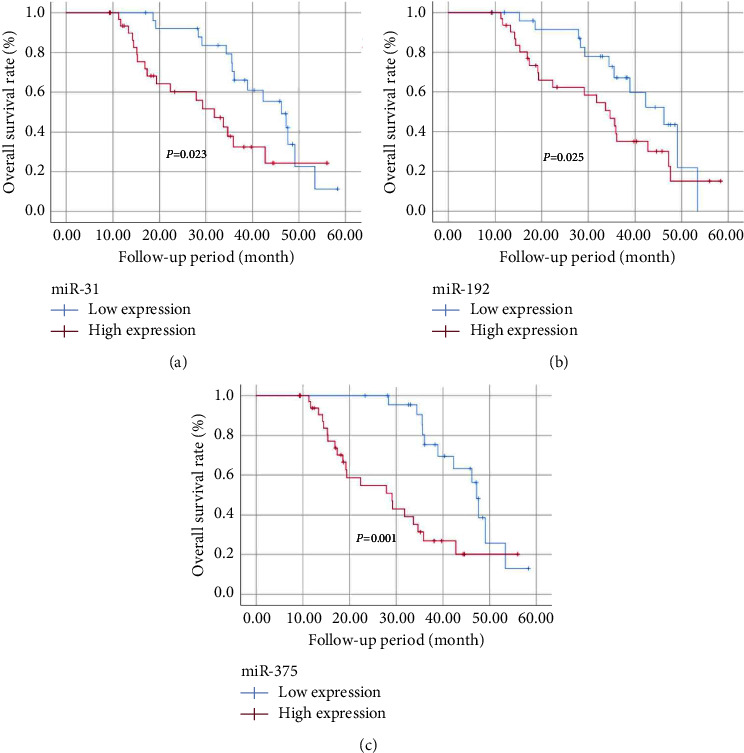
Kaplan–Meier curves of overall survival for GC patient based on exosomal miR-31 (a), miR-192 (b), and miR-375 (c) expression. Log-rank tests were applied for relatively low and high survival rates of patients according to the median of the ex-miRNA expression.

To evaluate the prognostic values of exosomal miR-31, miR-192, and miR-375, all GC patients were divided into two groups as mentioned above. Kaplan–Meier survival curves (Figure 4) exhibited that patients with low expression levels of exosomal miR-31, miR-192, and miR-375 appeared to have improved OS compared to those in the high expression group. In addition, Cox proportional risk regression model was performed to analyze the univariate and multivariate hazard ratios for OS. Age, gender, neural invasion, vascular invasion, TNM stage, and expressions of exosomal miR-31, miR-192, and miR-375 were, respectively, examined in a univariate analysis. A multivariate analysis was sequentially performed for variables which presented statistical significance in the univariate analysis. As Table 3 presents, TNM stage (*P*=0.003), exosomal miR-31 (*P*=0.026), and miR-375 levels (*P*=0.002) exhibited significance for OS in the univariate analysis, whereas the expression of ex-miR-375 (*P*=0.001) and TNM stage (*P*=0.001) remained significant in the multivariate analysis.

### 3.8. Bioinformatic Analysis of Ex-miRNAs

**Figure 5:**
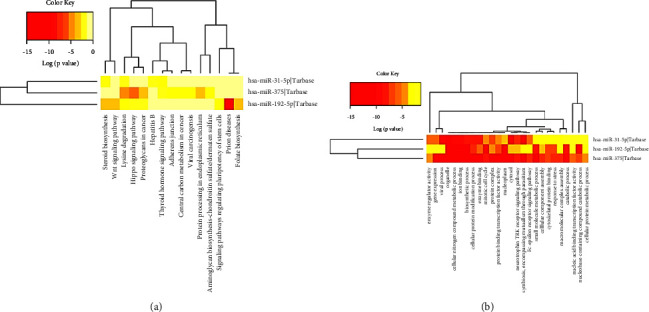
Heatmap of KEGG (a) and GO (b) analyses with the experimentally validated target genes of the three miRNAs. KEGG: Kyoto Encyclopedia of Genes and Genomes; GO: Gene Ontology.

A pathway analysis web server (DIANA-miRPathv7.0) including Kyoto Encyclopedia of Genes and Genomes (KEGG) and Gene Ontology (GO) analyses was applied to explore the underlying mechanisms of ex-miRNAs involved in tumorigenesis and progression. As Figure 5(a) shows, KEGG analysis demonstrated ex-miRNAs could play important roles in GC progression via several signaling pathways such as Wnt signaling pathway, Hippo signaling pathway, and proteoglycans in cancers. GO analysis (Figure 5(b)) indicated that the ex-miRNAs could serve as significant regulators in processes like gene expression, enzyme binding, and cellular protein metabolic process.

## 4. Discussion

Recently, more and more evidence has indicated that tumor cells release higher levels of exosomes than normal cells, which promote tumorigenesis through facilitating the crosstalk between tumor cells and other cell types [16]. It is well acknowledged that exosomes carry various kinds of cargos [3]. Analyses of exosomal cargos, especially ex-miRNAs, have attracted special attraction probably because of their stability and specific characteristics [17]. Multiple investigations have identified some ex-miRNAs participate in pathological changes and tumor progression by regulating oncogenesis, invasion, and metastasis [18]. These facts suggested that specific ex-miRNAs could act as promising diagnostic and predictive indicators in human cancers [17]. Recent studies have evaluated the clinical values of several ex-miRNAs in cancers such as ESCC [4], prostate cancer [11], and lung cancer [12]. However, there is little research analyzing the relationship between exosomal miRNAs and clinical significance in GC patients. The current study was conducted to identify specific ex-miRNAs as diagnostic and prognostic biomarkers for GC.

To select differentially expressed ex-miRNAs in GC patients from those in healthy controls, we combined the dbDEMC database and our miRNA microarray data. Exosomal miR-31, miR-192, and miR-375 were detected to be three of the most significantly upregulated miRNAs. Thus, we evaluated miR-31, miR-192, and miR-375 from several candidate miRNAs and revealed that the expression levels of these three ex-miRNAs were closely related to the detection and prediction of GC.

It is complicated to identify the roles of miR-31 in tumors, as numerous studies have reported that the expression level and function of miR-31 vary according to the cancer types. It could serve as a potential carcinogenic factor in lung cancer [19], colorectal cancer [20],and oral cancer [21]. However, in breast cancer [22], ovarian cancer [23], and prostate cancer [24], miR-31 is markedly downregulated. Different levels of miR-31 expression might result from the different organs of origin and the expression levels of miR-31 were tissue-specific.

A mechanism underlying the antitumor effects of miR-31 has been clarified in triple negative breast cancer (TNBC) tissues and cell lines [25], in which miR-31 directly targeted protein kinase C (PKC) in the NF-*ҝ*B signaling pathway, while in prostate cancer cell lines [24], miR-31 indirectly controlled the PI3K/AKT signaling pathway via targeting integrins and inhibited several metastasis-promoting genes such as RhoA and RDX [14] to repress tumor invasion and migration. Furthermore, it has been reported that miR-31 could modulate the p53 signaling pathway, suppressing the proliferation of serous ovarian cancer cells [14]. On the contrary, miR-31 can also function as an oncogenic microRNA to promote tumorigenesis and progression. For example, in colorectal cancers, the overexpression of miR-31 was implied to accelerate tumor proliferation through activating the RAS pathway and regulate the expression of RAS p21 GTPase activating protein 1 (RASA1) at a posttranscriptional level [26]. The oncogenic role of miR-31 has also been reported in non-small-cell lung carcinoma (NSCLC). The overexpression of miR-31 was found to be positively associated with KRAS, a key component participating in lung epithelial cell transformation, tumorigenesis, and epithelial mesenchymal transition (EMT) [25].

It was reported that miR-31 was downregulated in GC tissues and cell lines and acted as a tumor suppressor in GC, by affecting apoptosis, cell cycle, and cancer invasion [14]. Conversely, our study found that exosomal miR-31 in the plasma samples was highly expressed in GC patients compared to health controls. We attributed this inconsistency to the different sample origin. The lack of correlation between the expression of some miRNAs in exosomes and tissue samples [27, 28] or cell lines [20] has been verified in previous studies. For example, the let-7 miRNA expression is different in the intracellular level and exosomal level [29]. It was speculated that cancer cells release specific miRNAs into the extracellular environment in a selective and active manner through exosomes. In the current research, the univariate analysis for OS based on a Cox proportional risk regression model revealed that the lower the ex-miR-31 level, the longer the survival time. However, multivariate analysis found no statistical difference between the expression of ex-miR-31 and the overall survival time.

MiR-192 is considered to be a risk factor for migration, growth, and metastasis in some specific cancers, such as non-small-cell lung cancer (NSCLC) [15] and esophageal cancer [30]. Kakugawa et al. and his colleagues demonstrated that miR-192 silenced an atypical pathway inhibitor called NOTUM, which drives the Wnt signaling pathway [31]. In addition, miR-192 was found to upregulate FOXM1, through which the expression level of Wnt pathway could be increased [32]. Moreover, a significant correlation between miR-192 and cancer cell immune evasion mechanisms has been reported. miR-192 seemed to suppress some specific tumor antigens, including MAGE-A2 and MAGE-D4B, which involved in the regulation of the tumor microenvironment and immune response [33]. It is worth noting that, Chen et al. reported the level of circulating miR-192 in GC patients with distant metastasis was significantly higher than in patients with benign gastric ulcer and chronic gastritis. In consistence with previous studies, our data found that exosomal miR-192 was significantly upregulated in GC patients. Besides, we also found that ex-miR-192 was expressed in higher level in male than in female patients. Last but not the least, KEGG analysis confirmed that exosomal miR-192 was closely associated with the Wnt signaling pathway in GC.

miR-375 has been reported as a multifunctional microRNA. Clinical values of circulating miR-375, but not ex-miR-375, have been assessed in a variety of cancer types, including prostate cancer [21], hepatocellular carcinoma (HCC) [34], esophageal carcinoma (EC) [35], melanoma, and glioma [36, 37]. Chang et al. found lower expression of serum miR-375 in HCC cells and tissues and identified its antitumor effects in autophagy and cell viability. It plays a repressive role by inhibiting ATG7, an essential autophagy-associated gene [34]. Also, Komatsu et al. reported downregulation of plasma miR-375 in EC patients and the positive association between the expression level of miR-375 and overall survival [35]. Overall, miR-375 is downregulated in several human cancers and acts as a tumor suppressor.

Accumulated investigations have focused on miRNA-375 derived from exosomes. Multiple evidence found that expressions of exosomal miR-375 increased in numerous cancers such as locally advanced rectal cancer (LARC) [38], late-stage prostate cancer [21], and small cell lung cancer (SCLC) [39]. Upregulation of ex-miR-375 leads to numerous biological changes, including the suppressed activity of antigen-presenting dendritic cells (DC) [40], enhanced immunosuppressive environment [41], and inflammatory and neoangiogenic responses [42], as often seen in advanced or metastatic cancers. Interestingly, miR-375 was found to be downregulated in GC tissues and could suppress tumor cell proliferation through Janus kinase 2 (JAK2) inhibition [43]. Also, Tzivion et al. observed miR-375 could function as an antitumor factor for GC via targeting YWHAZ, an oncogene associated with cell survival signaling, cell cycle, and apoptosis [44]; while our data demonstrated the exosomal miR-375 significantly upregulated in GC patients compared to negative controls, we also found that a high expression level of ex-miR-375 was associated with poor OS in GC patients. An explanation on the inconsistency might account for the population discrepancy, different environments, and small sample sizes. Univariate and multivariate analyses in the current research both revealed that the expression level of ex-miR-375 was an independent prognostic factor in GC.

The reasons why we preferred exosomal miRNAs isolated from blood samples rather than those from cancer tissues or circulating cells are as follows: First, exosomes are widely found in body fluids, including blood, tears, urine, and saliva, which makes them promising in tumor diagnosis and treatment. Detection of plasma ex-RNAs is minimally invasive without the need for a biopsy or surgically resected tissue, making it an ideal “liquid biopsy” method. Second, it can be repeatedly performed at relatively low cost and provide clinical information during the course of treatment with convenience. Third, compared to other methods, qRT-PCR is more quantitative and sensitive. Last but not the least, different changes of miRNA expressions are detected in exosomes and plasma samples, indicating a distinct mechanism underlying the packaging of miRNAs into exosomes or other carriers. We hypothesized that the discrepancy was partially due to the instability of miRNAs in plasma. Exosomes could protect miRNAs from degradation [45] and this is the reason why we prefer miRNAs in exosomes for our research rather than in the serum or plasma. Xie et al. detected miRNAs from plasma and exosomes in Sprague–Dawley (SD) rats and compared the differences. A specific subset of miRNA enriched in exosomes was found [46].

There are several limitations remained in this research. First, the mechanisms via which exosomal miRNAs modulate cancer processions are not clear. Second, the number of patients is small, and no adjacent samples are analyzed. Also, potential election bias exists, as in all clinical studies. Since expression levels of ex-miRNAs might vary according to the sample origins, further studies are needed to explain the contradiction and provide recommendations of candidate biomarkers for GC patients. Last but not the least, we only evaluated the baseline expression levels of exosomal miR-375, miR-192, and miR-31. It is necessary to establish a follow-up program comprising ex-miRNA levels, other blood chemistry methods, and computed tomography (CT) to further discover the underlying association between the ex-miRNA expressions and tumor progression.

Taken together, we observed the upregulation of exosome-derived miR-375, miR-192, and miR-31 in GC patients. They were significantly related to pathologic findings and clinical outcomes. This study indicated that exosomal miRNAs could be considered to be robust candidates of clinical diagnostic and prognostic biomarkers for GC patients. Prospective validation studies should be conducted to support our findings.

## Figures and Tables

**Table 1:** Characteristics of 60 GC patients and 63 healthy controls.

Variables	Cases (%)	Controls (%)
Number	60	63

*Gender*		
Male	40 (66.7)	46 (73)
Female	20 (33.3)	17 (27)

*Age*		
<65	32 (53.3)	37 (58.7)
≥65	28 (46.7)	26 (41.3)

*Location*		
Proximal	13 (21.7)	
Middle	26 (43.3)	
Distal	21 (35)	

*Differentiation*		
Well/moderately	24 (40)	
Poorly	24 (40)	

*Lauren classification*		
Intestinal	13 (21.7)	
Diffuse	14 (23.3)	
Mixed	16 (26.7)	

*Neural invasion*		
Yes	19 (31.7)	
No	35 (58.3)	

*Vascular invasion*		
Yes	22 (36.7)	
No	32 (53.3)	

*TNM stage*		
I	12 (20)	
II	12 (20)	
III	30 (50)	
IV	6 (10)	

**Table 2:** The association of the miRNAs with clinical features in GC patients (presented as *P* value).

miRNA	Age	Gender	Location	Differentiation	Lauren classification	Neural invasion	Vascular invasion	TNM stage
miR-31	**0.008**	0.754	0.134	0.523	0.724	0.463	0.388	0.909
miR-192	**0.005**	**0.036**	0.288	0.704	0.666	0.616	0.736	0.322
miR-375	**0.05**	0.339	0.152	0.364	0.707	0.593	0.765	0.825

The significance of bold values represents that *P* value is greater than or equal to 0.05.

**Table 3:** Univariate and multivariate cox analyses for OS in GC patients.

Variables	*Univariate analysis*		*Multivariate analysis*	
	HR (95% CI)	*P* value	HR (95% CI)	*P* value
*Age*	0.753 (0.356–1.592)	0.458		
<60 vs. ≥60 years				

*Gender*	0.903 (0.432–1.886)	0.786		
Male vs. female				

*Vascular invasion*	0.718 (0.358–1.438)	0.350		
Absent vs. present				
*TNM stage*	0.281 (0.120–0.655)	0.003	0.174 (0.071–0.425)	<0.001
I + II vs. III + IV				

*Neural invasion*	0.742 (0.373–1.476)	0.395		
Absent vs. present				
*Ex-miR-31 expression*	0.447 (0.220–0.908)	0.026	1.892 (0.561–6.377)	0.304
Low vs. high				

*Ex-miR-192 expression*	0.555 (0.272–1.130)	0.104		
Low vs. high				
*Ex-miR-375 expression*	0.306 (0.146–0.638)	0.002	0.104 (0.028–0.389)	0.001
Low vs. high				

## Data Availability

All experimental data used to support the findings of this study are available from the corresponding author upon request.

## Supplementary Materials

Supplementary Materials Here are two expression heatmaps downloaded from the Database of Differentially Expressed MiRNAs in Human Cancers (dbDEMC). MiR-31, miR-375 (Supplementary 1), and miR-192 (Supplementary 2) have different expression levels in “cancer vs normal” signatures. Red boxes represent significant upregulation in cancers compared to normal tissues, green boxes represent significant downregulation in cancers compared to normal tissues, and white boxes represent no significance or missing data.
