# Supplementary figures and images for: Exosome-Encapsulated miR-31, miR-192, and miR-375 Serve as Clinical Biomarkers of Gastric Cancer
Source: J Oncol. 2023 Feb 16;2023:7335456. doi: 10.1155/2023/7335456 (PMC9950326; doi:10.1155/2023/7335456)

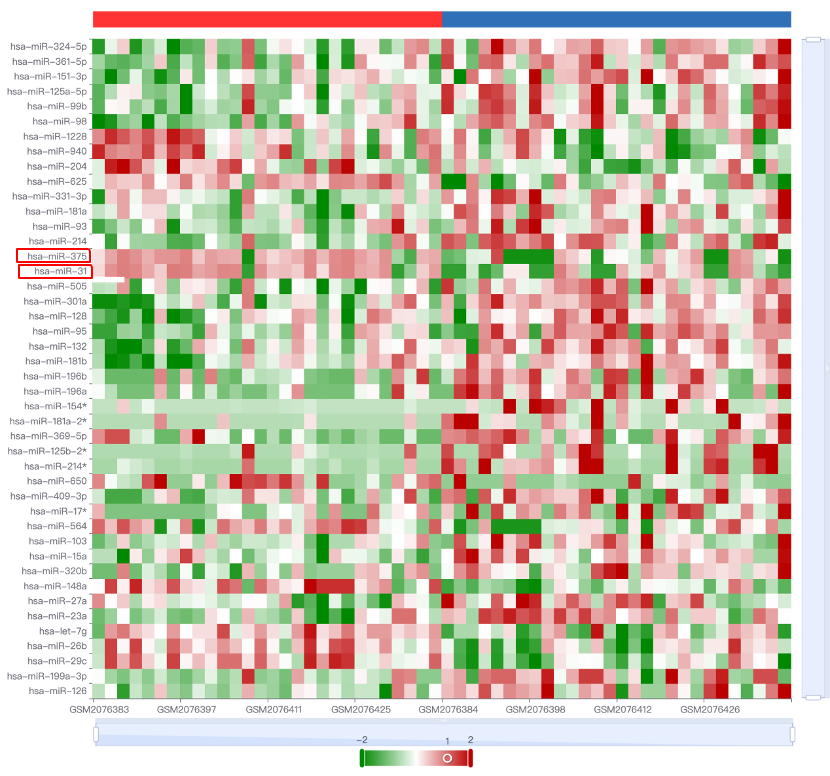

Supplement: Supplementary Materials — Here are two expression heatmaps downloaded from the Database of Differentially Expressed MiRNAs in Human Cancers (dbDEMC). MiR-31, miR-375 (Supplementary 1), and miR-192 (Supplementary 2) have different expression levels in “cancer vs normal” signatures. Red boxes represent significant upregulation in cancers compared to normal tissues, green boxes represent significant downregulation in cancers compared to normal tissues, and white boxes represent no significance or missing data. [file 7335456.f1.zip › Supplementary A.jpg]

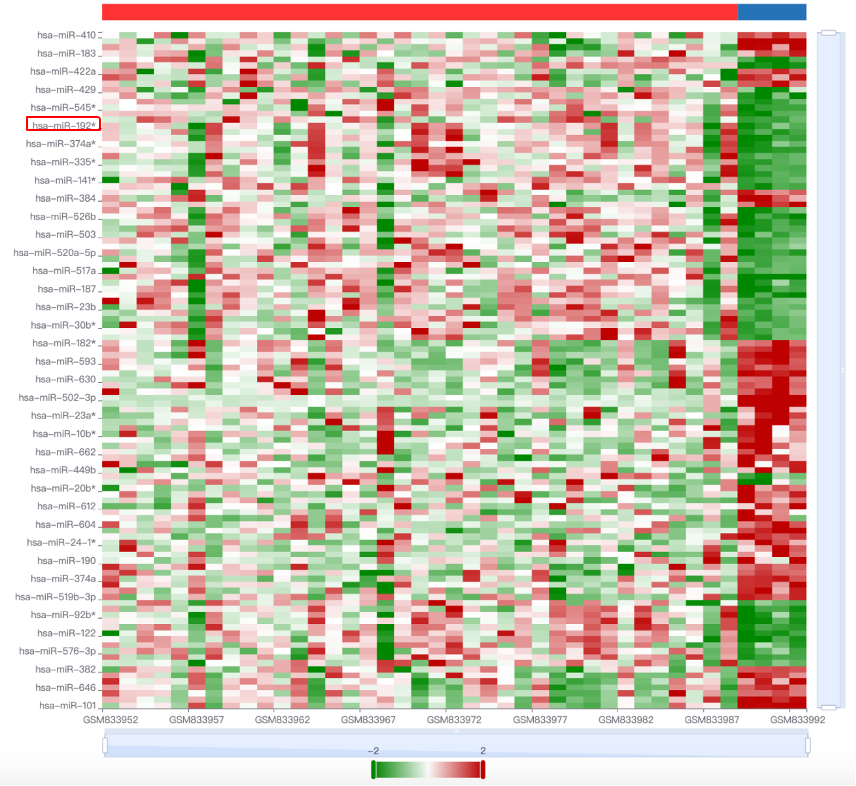

Supplement: Supplementary Materials — Here are two expression heatmaps downloaded from the Database of Differentially Expressed MiRNAs in Human Cancers (dbDEMC). MiR-31, miR-375 (Supplementary 1), and miR-192 (Supplementary 2) have different expression levels in “cancer vs normal” signatures. Red boxes represent significant upregulation in cancers compared to normal tissues, green boxes represent significant downregulation in cancers compared to normal tissues, and white boxes represent no significance or missing data. [file 7335456.f1.zip › Supplementary B (1).jpg]
